# Entropy Reduction Across Odor Fields

**DOI:** 10.3390/e27090909

**Published:** 2025-08-28

**Authors:** Hugo Magalhães, Lino Marques

**Affiliations:** Department of Electrical and Computer Engineering, Institute of Systems and Robotics, University of Coimbra, 3030-290 Coimbra, Portugal; hugo.magalhaes@isr.uc.pt

**Keywords:** mobile robotics, odor source localization, cognitive decision-making, entropy

## Abstract

Cognitive Odor Source Localization (OSL) strategies are reliable search strategies for turbulent environments, where chemical cues are sparse and intermittent. These methods estimate a probabilistic belief over the source location using Bayesian inference and guide the searching movement by evaluating expected entropy reduction at candidate new positions. By maximizing expected information gain, agents make informed decisions rather than simply reacting to sensor readings. However, computing entropy reductions is computationally expensive, making real-time implementation challenging for resource-constrained platforms. Interestingly, search trajectories produced by cognitive algorithms often resemble those of small insects, suggesting that informative movement patterns might be replicated using simpler, bio-inspired searching strategies. This work investigates that possibility by analysing spatial distribution of entropy reductions across the entire search area. Rather than focusing on searching algorithms and local decisions, the analysis maps information gain over the full environment, identifying consistent high-gain regions that may serve as navigational cues. Results show that these regions often emerge near the source and along plume borders and that expected entropy reduction is strongly influenced by prior belief shape and sensor observations. This global perspective enables identification of spatial patterns and high-gain regions that remain hidden when analysis is restricted to local neighborhoods. These insights enable synthesis of hybrid search strategies that preserve cognitive effectiveness while significantly reducing computational cost.

## 1. Introduction

Odor Source Localization (OSL) is the process of finding the origin of a chemical release event by reasoning with observed data [1]. This is extremely relevant for emergency scenarios such as search and rescue operations or in the event of chemical spills which require fast interventions by locating the source of dispersing harmful substances. Volatile chemicals released into the environment are transported by advective flow and mixed with the main fluid through turbulence and diffusion. This process gives rise to a dispersed concentration structure known as a chemical plume [2]. In natural scenarios, fluid instabilities combined with the shape of the environment further fragment the plume into irregular patches, disrupting what could be a smooth concentration gradient by an intermittent and fluctuating pattern [3].

Estimating the structure of a plume and locating its source from sparse sensor readings poses a complex inverse problem [4,5]. A common approach to this challenge formulates it from an information-theoretic perspective [6,7], where the agent maintains a belief map representing the probability distribution over possible source locations. Initially uniform, this distribution is iteratively updated as new observations are acquired. To set this process, it is common to discretize the space with grid cells and update the probability of each cell containing the source. Shannon entropy [8,9] quantifies the uncertainty of this belief, and the objective becomes to reduce entropy over time through informative measurements. Cognitive search strategies operationalize this concept by selecting actions that maximize the expected reduction in entropy [10]. These methods integrate past observations via Bayesian inference and guide agents toward regions likely to yield informative measurements. While effective in turbulent and sparse signal environments, the estimation of expected information gains is computationally intensive. For instance, evaluating expected information gains across *n* possible actions requires O(n×k×N) computations per decision step, where *k* represents potential observations and *N* the number of grid cells, making real-time implementation prohibitive for large action spaces. As a result, practical implementations often restrict decisions to a limited set of candidate positions, potentially leading to sub-optimal trajectories and delayed convergence.

A relevant question within this framework is where the locations are expected to yield the highest information gains. Since these gains depend on current belief state, search direction and efficiency are highly influenced by the agent’s knowledge and acquired measurements [11]. Initially, uniform belief leads to broadly exploratory behaviours, whereas later stages favor exploitation near high posterior probability regions. This adaptive sampling strategy is essential for maintaining search balance. Despite complexity, cognitive methods generate search trajectories resembling those of small insects [12,13]. For example, silkworm moths exhibit crossflow zigzag patterns when losing plume contact and surge upflow when detecting odorants [14]. These behaviours remarkably mirror exploration and exploitation strategies observed in cognitive algorithms despite moths lacking capacity for Bayesian inference and entropy calculations. Reactive and bio-inspired approaches are easily implemented with low computational requirements, however, they rely on tunable parameters depending on target scenarios. This raises the question of whether advantageous aspects from bio-inspired and cognitive approaches can be combined to synthesize hybrid methods with high performance and moderate computational costs.

This work addresses the above question by examining cognitive decision-making through a spatial analysis of entropy reductions, with the goal of identifying high gain regions that could inform the design of more efficient localization strategies. Unlike conventional approaches that focus on the searching algorithm and evaluate the expected information gain at a local and limited set of admissible actions near the agent, this study analyzes the actual entropy reduction across the entire search space.The study comprises four main objectives: (1) assess how measurement positions influence the shape and evolution of the belief map, (2) investigate how the prior belief affects the spatial distribution of future information gains, (3) analyze the spatial distribution of entropy reductions with a focus on plume structures and (4) quantify the diversity of entropy reductions using a spatial entropy measure to determine the dependence of spatial configurations on the total entropy value. The results show that the shape of a chemical plume plays a critical role in determining cognitive decisions, with high-interest (gain) regions frequently emerging across the plume borders and in proximity to the source. Furthermore, the agent’s belief significantly affects decision-making with high-uncertainty conditions originating high-gain regions that tend to produce cross-flow behaviours, while lower-uncertainty beliefs lead to more directed movements with high-gain regions localized within the active plume area and at source proximity. These findings support developing hybrid OSL algorithms that replicate cognitive agent tendencies by following informative regions while performing search behaviors with lower computational demands, such as bio-inspired strategies.

## 2. Problem Formulation

Consider a mobile agent moving in known locations $p=\left(x,y\right)\in R^{2}$ with the capability to sense for chemical measurements c(p,t) at time step t≥0 s. Furthermore, assume a bounded workspace Ω with fluid flowing in a dominant direction $\vec{u}$ aligned with the *x* axis and a source in an unknown position $\left(x_{s},y_{s}\right)$ releasing a chemical pollutant at an unknown rate *Q*. It is assumed that the source and measurement height are at the same level, restricting the search to a 2D space. The agent searches for the source using a cognitive OSL algorithm that consists of two phases: (1) belief update: the agent maintains a probabilistic belief over the source location, which is updated sequentially using new concentration observations and (2) decision making: at each time step, the agent evaluates the expected information gain (i.e., reduction in uncertainty) at a set of admissible movement positions, selecting the one that maximizes this expected gain. The goal here is to study the spatial distribution of expected uncertainty reductions in order to assess the influence of the plume structure and prior belief maps on information gains and to identify high-interest (gain) regions that can prove to be valuable for the design of new OSL algorithms.

## 3. Methods

### 3.1. Plume Model

Dispersion models are fundamental tools to reason about the olfactory observations that a robot acquires along its mission. This work adopts a Gaussian plume model (1) which provides an analytical solution of the advection–diffusion equation for a point source, located at position $\left(x_{s},y_{s},h\right)$ in a Cartesian coordinate frame, releasing at constant rate *Q*, with average fluid $\bar{u}$ flowing steadily with a speed $u_{s}$ in the direction $u_{d}$ and assuming an infinite space.

(1) $$\begin{array}{c} M=\frac{Q}{2\pi \bar{u}\sigma_{y}\sigma_{z}}\exp\left(\frac{-y^{2}}{2\sigma_{y}^{2}}\right)\left[\exp\left(\frac{-{\left(z-h\right)}^{2}}{2\sigma_{z}^{2}}\right)+\exp\left(\frac{-{\left(z+h\right)}^{2}}{2\sigma_{z}^{2}}\right)\right] \end{array}$$

The lateral and vertical dispersion coefficients, $\sigma_{y}$ and $\sigma_{z}$, respectively, are functions of the downwind distance (*x*) to the source, modeled as $\sigma_{y}^{2}=2\cdot D_{y}\cdot \left(x-x_{s}\right)/u_{s}$ and $\sigma_{z}^{2}=2\cdot D_{z}\cdot \left(x-x_{s}\right)/u_{s}$.

### 3.2. Bayesian Inference

To continuously update the belief about the potential source location, observed data are incorporated using Bayesian inference, which plays a central role in guiding the movement decisions of the searching agent. As in [15], the posterior probability density function (PDF) of the state vector $\theta_{t}$ given a set of concentration measurements $c_{t}^{1:k}$ is estimated as

(2) $$P\left(\theta_{t}|c_{t}^{1:k}\right)\propto P\left(c_{t}^{1:k}|\theta_{t}\right)P\left(\theta_{t-1}\right)$$

In this formulation, the state space θ includes the source parameters such as the 2D source location and plume characteristics, typically $\theta =[x_{s},y_{s}]$, and optionally including parameters like *Q*, $D_{y}$, and $D_{z}$. In the grid-based approach used in this work, the belief is represented as a discrete probability distribution over a finite grid of source hypotheses, where each cell *j* corresponds to a candidate source location and stores a probability $P{\left(j\right)}_{t}$ such that $\sum_{j=1}^{N}P{\left(j\right)}_{t}=1$, where *N* represents the total number of cells. At each time step, the prior belief $P(\theta_{t-1})$ is updated by incorporating the new set of observations. For each grid cell (i.e., each hypothesis $\theta_{t}^{j}$), the likelihood of the observation given that hypothesis is computed using a Gaussian model:

(3) $$P\left(c_{t}^{1:k}|\theta_{t}^{j}\right)\propto \exp\left(-\frac{1}{2}\sum_{i=1}^{k}\left(\frac{{\left(c_{i}-M\left(p_{i},\theta_{t}^{j}\right)\right)}^{2}}{\sigma_{d}^{2}}\right)\right)$$

Here, *k* is the number of measurements obtained up to time *t*, $p_{i}$ is the position of the *i*-th observation, $M(p_{i},\theta_{t}^{j})$ is the predicted concentration at $p_{i}$ using the Gaussian plume model with source hypothesis $\theta_{t}^{j}$ and $\sigma_{d}^{2}$ denotes the variance of measurement errors proportional to the modeled concentration. After computing the likelihoods for all grid cells, the belief is updated via Bayes’ rule by re-normalizing:

(4) $$P\left(\theta_{t}|c_{t}^{1:k}\right)=\frac{P\left(c_{t}^{1:k}|\theta_{t-1}^{j}\right)\cdot P\left(\theta_{t-1}^{j}\right)}{\sum_{j=1}^{N}P\left(c_{t}^{1:k}|\theta_{t-1}^{j}\right)\cdot P\left(\theta_{t-1}^{j}\right)}$$

This grid-based inference approach provides a spatially explicit and interpretable representation of uncertainty.

### 3.3. Cognitive Movement Decision

The decision process guides the agent toward the most informative positions, defined as those that minimize the expected uncertainty about the source location, quantified with the so-called information utilities [16]. At each admissible future position *u*, the expected gain in information is computed as the difference between the entropy of the current belief map and the expected entropy obtained from a future belief map updated with a hypothetical observation at that position. The actual uncertainty is computed from the belief map at time step *t* using Shannon’s entropy $S_{t}$.

(5) $$S_{t}\approx -\sum_{j=1}^{N}P{\left(j\right)}_{t}\log\left(P{\left(j\right)}_{t}\right)$$

To compute the expected entropy, a discrete set of $N_{z}$ potential observations $z_{1},z_{2},\ldots ,z_{m}$ is taken. Each observation $z_{m}$ is associated with a probability of occurring, denoted as $P(z_{m}|\theta_{t})$, which is estimated using the plume dispersion model and the actual belief. For each potential observation $z_{m}$ a future belief map $\theta_{t+1}$ is computed using Bayes’ rule (Equation (2)), followed by its entropy computed with Equation (5). The total expected entropy is obtained by weighting each future entropy with its corresponding observation probability:

(6) $$E\left[S_{t+1}\right]=\sum_{m=1}^{N_{z}}P\left(z_{m}|\theta_{t}\right)\cdot S_{t+1}^{m}$$

The expected uncertainty reduction at each possible movement location is then determined from the difference between the entropy of the actual belief map and the expected (future) entropy value as in

(7) $$E^{u}=S_{t}-E{\left[S_{t+1}\right]}^{u}$$

The agent selects the position *u* that yields the highest expected uncertainty reduction, which is then used as the next movement goal.

### 3.4. Spatial Analysis of Entropy Reduction

Consider a simplified case where the agent has no model of the environment, no sensor to measure chemical concentrations, and the source is situated in one of the cells. The belief starts uniform with each cell having a probability P(j)=1/N of containing the source, resulting in a maximum entropy value of $S_{max}=\log N$.

**Figure 1:**
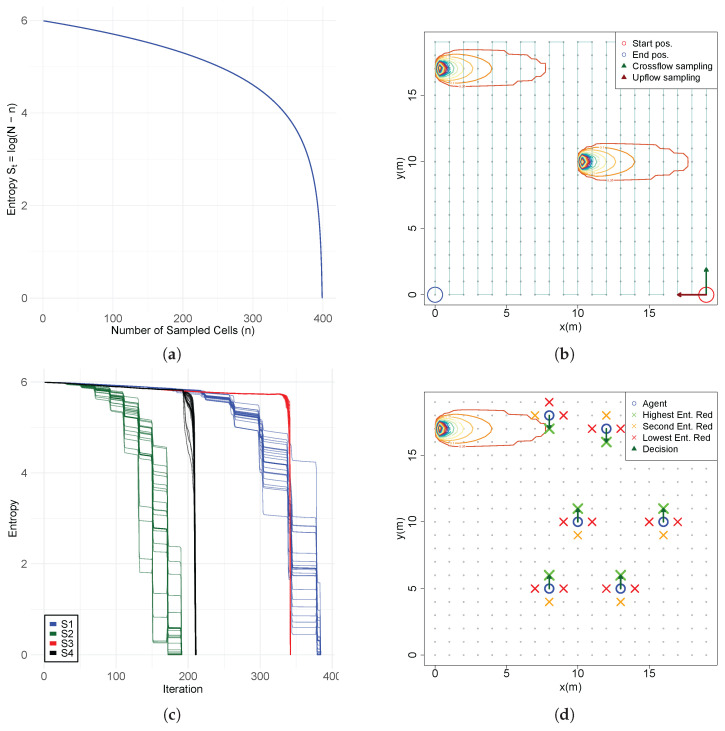
(**a**) Entropy reduction in a scenario without a dispersion model and sensor observations; (**b**) systematic sampling trajectory and plume active region; (**c**) entropy reduction in scenarios with dispersion model and sensor observations; (**d**) cognitive decision-making at multiple locations.

The agent samples each cell sequentially, setting the probability to zero when the source is not found and renormalizing the remaining probabilities, with the sum of all values equaling one. The belief distribution becomes increasingly concentrated in the unsampled cells but remains uniform although over fewer cells with the entropy decreasing with a logarithmic decay (Figure 1a) following the equation:

(8) $$S_{n}=\log\left(N-n\right)$$

where *n* represents the cells already sampled. This means that entropy decreases slowly during the first iterations and more sharply as the number of remaining cells reduces. When the source is found, the probability is concentrated in a single grid cell with the value of 1 resulting in an entropy value $S_{final}=0$. While the computational demands are minimal, this approach is highly inefficient requiring direct exclusion of nearly all incorrect cells to converge on the true source location.

In contrast, a more informed approach such as Infotaxis leverages chemical concentration measurements in combination with a physical dispersion model, such as the Gaussian plume model, to update the belief map. Here, the entropy reduction evolves differently, where instead of explicitly eliminating cells, the agent observes values that are related to the source location through a probability distribution. The likelihood of each observation adjust the probabilities across many cells simultaneously, concentrating the belief around areas consistent with the observed data and the plume dynamics. This leads to faster and more informative entropy reduction, even when the true source cell is not directly observed.

However, in this situation, a closed-form expression of the entropy reduction cannot be obtained. Chemical observations are a function of the source parameters and spatial configuration, containing significant noise originating from multiple sources of uncertainty such as sensor performance and environmental conditions. The Bayesian inference process relies non-linearly on the prior belief and the likelihood function, which is determined by a probability distribution and the plume dispersion model. Also, the surprise of each observation, which depends on the spatial phenomena and the mismatch between the predicted and real measurements, significantly influences the evolution of entropy during the search, decreasing faster in more certain environments and slower when the disturbances are meaningful.

Assume an informed agent capable of estimating the belief distribution of the source location from chemical observations systematically samples each cell in a 20×20 grid (1 m resolution). These measurements are sequentially used to update the source belief. The agent begins at the bottom-right corner (red circle) and traverses the space in either a crossflow direction (green arrow) or upflow direction (red arrow), following a serpentine sampling pattern until it reaches the source (Figure 1b). The environment contains a Gaussian chemical plume, with the source located either in the top-left corner (0, 17) or at the center (10, 10) and flow aligned with the *x* axis. Scenarios S1 and S2 implement crossflow movement, corresponding to the corner source (S1) and central source (S2), respectively. Scenarios S3 and S4 follow upflow trajectories, with sources at the same respective locations. The analysis of Figure 1c reveals that crossflow trajectories (S1, S2) generally result in a faster entropy reduction compared to upflow movements (S3, S4). Notably, S1 shows a more rapid early decline than S3. However, S3 ultimately achieves lower entropy levels sooner, due to higher chemical concentrations encountered near the source. For centrally located sources, S2 consistently yields faster and more substantial uncertainty reduction than S4. These entropy dynamics are significantly affected by sensor noise, modeled as normally distributed, which introduces variability in the belief updates. This highlights the complexity of predicting entropy evolution, driven by the interplay among sensor inaccuracies, belief update processes, and the spatial structure of the chemical distribution.

Now consider a scenario in which the agent possesses a highly uncertain belief about the source location and employs cognitive decision-making by evaluating the expected entropy reduction at cardinal directions to choose its next movement position. With the source located in the top-left corner (0, 17), an analysis of expected information gain across the search space (Figure 1d) reveals that crossflow decisions consistently yield the highest expected entropy reduction, whereas upflow and downflow movements are comparatively less informative. These results corroborate the findings obtained from sequential sampling scenarios, reinforcing the conclusion that crossflow movement in the direction of the plume provides the most valuable information gain. This behavior aligns with patterns commonly observed in cognitive observation-based source localization (OSL) strategies, where agents intensively explore the environment perpendicular to the flow to increase information gain and improve the likelihood of plume interception.

This work aims to study entropy reduction in an environment containing a chemical plume, independently of any specific search strategy for source localization. In typical cognitive OSL implementations, agents evaluate expected information gain only at a limited number of admissible positions near their current location as in the previous example. This localized decision-making approach constrains the understanding of where high-gain regions truly lie within the environment. To address this limitation, the present study assumes complete freedom in the sampling process, computing entropy reduction (Equation (7)) at every point in the environment, with particular focus on the active plume region and its surrounding areas.

The study will start by analyzing how measurement positions impact the shape of the posterior probability belief and its associated uncertainty. In a second stage, gains of information are evaluated across the entire search space, followed by a focused analysis within the active region of the plume. An important subject of interest lies on comprehending how variations in the prior belief influence the spatial distribution of entropy reductions. A third stage consists on examining the borders of the active region, that exhibit distinct and structured patterns of information gains.

Applying these computations to all candidate future positions, the study produces a comprehensive spatial map of information gains. However, a limitation of classical entropy is its inability to consider the spatial structure since it only reasons the probability distribution, not how probabilities are arranged in space. As a result, spatially distinct distributions with identical probabilities yield the same entropy value. Furthermore, as the study will show, entropy reductions are not uniformly distributed across the search space due to the influence of the plume structure and odor dynamics while also being spatially correlated with nearby cells containing similar entropy variations. Hence, a final stage will consist of applying a spatial entropy framework proposed by Altieri et al. [17] on the entropy reduction map, which incorporates spatial relationships into entropy computation. The goal is to investigate the diversity of information gains and quantify their dependence on spatial distributions. This enables a more nuanced analysis of how entropy reduction varies spatially, allowing us to better understand how it shapes movement decisions in cognitive searches.

### 3.5. Evaluation Process

The belief is discretized using a grid resolution of 0.25 m, while the entropy difference map is configured with a lower resolution of 0.5 m. A circular region with a 2 m diameter centered on source position is employed to avoid entropy reduction calculations on top of the source. The detection threshold is defined as 0.1 μg/m^3^. For a comprehensive analysis, in this study real entropy reductions are computed instead of the expected values; i.e, a single measurement is taken from the real plume model at each admissible action, assuming a probability of detection equaling to 1.

**Figure 2:**
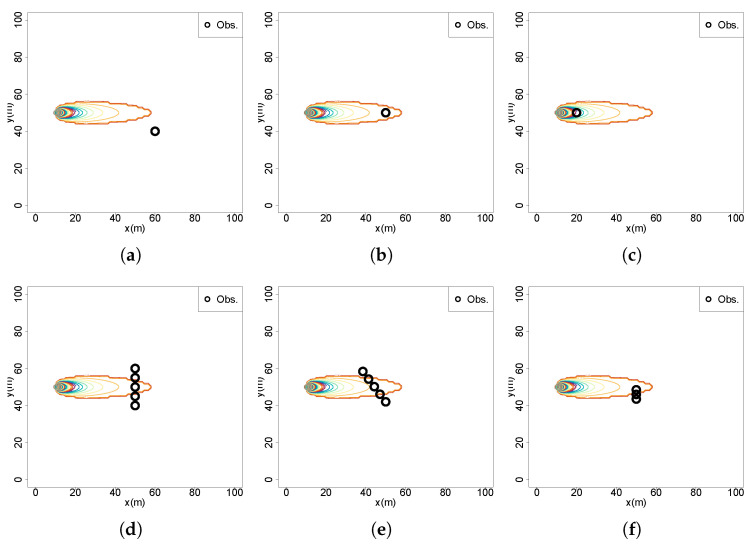
Measurement positions of the tested scenarios: (**a**) S2; (**b**) S3; (**c**) S4; (**d**) S5.2; (**e**) S7; (**f**) S8.

Since spatial entropy measurement requires categorical data, the continuous entropy differences are classified into eight equally separated intervals, between the minimum and maximum entropy (denoted between “E1” and “E8”). From the literature, smaller co-occurrence distances are deemed most relevant; therefore, partial mutual information and partial residual entropy are computed for five distance intervals, namely [0,1], [1,2], [2,3], [3,5] and [5, dmax], where dmax denotes the maximum diagonal distance of the square searching region. These distances support analysis on both local scales, as in cognitive decision-making, where the agent assesses nearby information gains and global scales which encompass the entire searching area. A significant number of testing scenarios are evaluated with the prior belief updated with the following.

(S1) Initial uniform belief with equal probability across all cells (1/N). No observations are taken;
(S2) A single observation taken far from the source, at x=60, y=40, below the detection threshold (Figure 2a);
(S3) A single observation taken far from the source, at x=50, y=50, above the detection threshold with a concentration value of c=0.2387324 (Figure 2b);
(S4) A single observation taken near the source, at x=20, y=50, above the detection threshold with a concentration value of c=0.9549297 (Figure 2c);
(S5) A sequence of observations across the active region and far from the source x=50, with three (S5.1), five (S5.2 Figure 2d) and ten (S5.3) observations;
(S6) A sequence of observations with two crossings over the active region and far from the source, x=40 and x=50;
(S7) A diagonal sequence of five observations across the active region, starting at x=50, y=40 with an angle 125deg relative to the flow (Figure 2e);
(S8) Measurements obtained across plume borders y=[41.5,44,46.5] at a downflow distance x=50 (Figure 2f).

**Figure 3:**
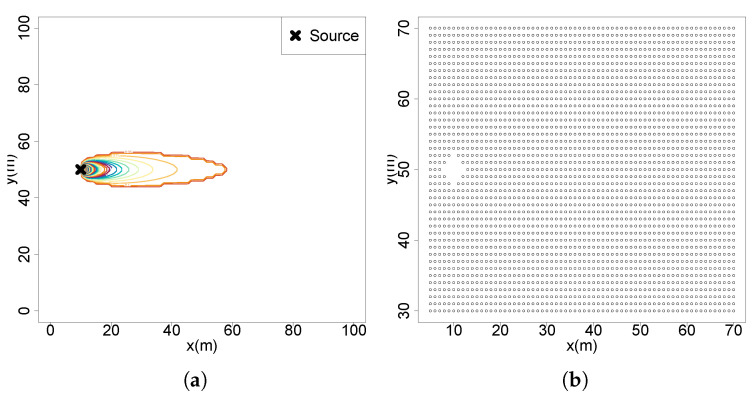
(**a**) Testing environment and Gaussian plume; (**b**) entropy reduction grid.

This study is performed in a simulated searching area with 100 m × 100 m dimensions and is discretized into a 2D grid of 0.25 m making a total of 100,000 N positions. The GPM is used to generate a plume with fixed parameters that is approximately 1/10 of the searching area with the flow moving in the direction of the x axis with a speed of 1.0 m/s, emission rate *Q* of 30 g/s and dispersion parameters $\sigma_{y}$ and $\sigma_{z}$ equal to 0.5. The testing environment and the positions of future gains of information are shown in Figure 3a and Figure 3b, respectively. The simulations are designed with R programming language (version 4.5.1) and processed in a computer equipped with a Ryzen 7 5700x processor, 32 GB DDR4 RAM and a Nvidia GTX1660 graphics card.

## 4. Experimental Results

### 4.1. Belief Maps Obtained from Different Measurement Positions

**Figure 4:**
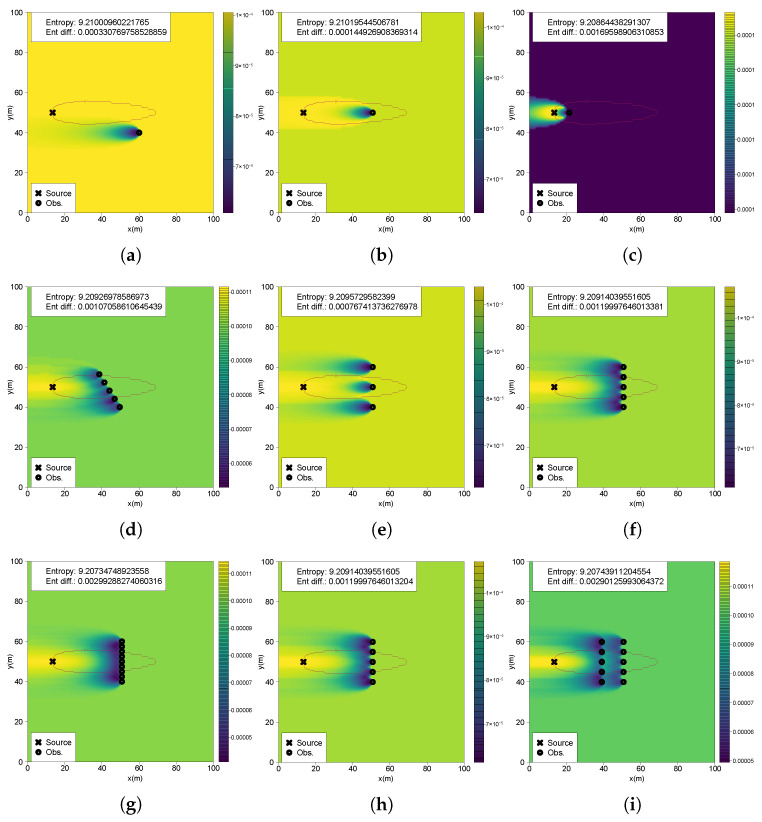
Belief maps inferred from observations at each tested scenario: (**a**) S2; (**b**) S3; (**c**) S4; (**d**) S7; (**e**) S5.1; (**f**) S5.2; (**g**) S5.3; (**h**) S5.2 with all measurements directly assimilated; (**i**) S6.

Scenario 1 starts with a uniform belief map without observations, yielding maximum entropy (9.21034 from log(N)). In Scenario 2 (Figure 4a), a single observation far from the source and below the detection threshold produces a minimal entropy reduction (0.00033). The resulting belief map exhibits a low-probability region near the measurement point, shaped as an inverse Gaussian plume, consistent with the likelihood model using a Gaussian plume and normal distribution. Consequently, the probability increases gradually in the upflow direction, dispersing uniformly in the remaining regions. Scenario 3 (Figure 4b), with a single observation slightly above the detection threshold, presents a distinct probability distribution. An inverse plume shape originates from the measurement location, with probabilities increasing significantly beyond a certain distance upflow, differentiating this scenario from Scenario 2. Despite this distinct shape, entropy reduction remains relatively low, confirming previous findings of limited information gain from distant locations of the source. Scenario 4 (Figure 4c) performs an observation close to the source with a high chemical reading, resulting in a greater entropy reduction (0.001695). Although this yields a concentrated high-probability region towards the source, uncertainty remains high due to numerous potential source locations.

**Figure 5:**
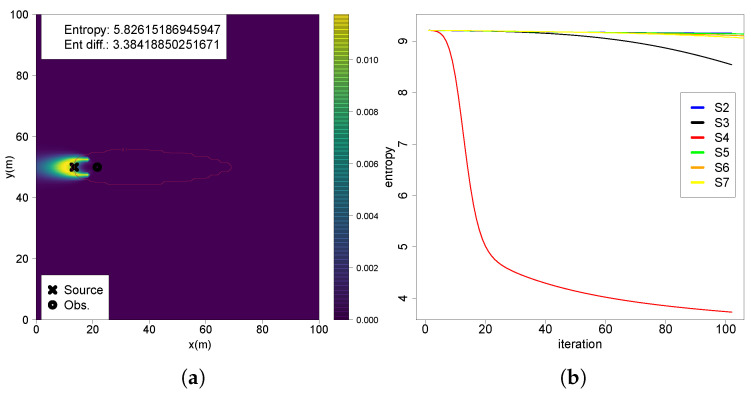
(**a**) Belief map S4 sequentially updated multiple times with the same observation; (**b**) entropy of belief sequentially updated multiple times with the same observation at each scenario.

Repeatedly updating the belief with this observation substantially decreases uncertainty, as shown by a entropy reduction of 3.384 (Figure 5a). The entropy reduction along multiple iterations shows a steep decline after a few iterations (Figure 5b), concentrating even more high-probability regions near the true source position. However it does not provide the same results for other beliefs, specially for the ones updated with measurements far from the source, with entropy remaining high for a large number of iterations.

Scenario 5 shows that entropy reduction increases with the number of observations, which is expected from the findings of Scenario 4. Three observations yielded a reduction of 0.0007674 (Figure 4e), five yielded 0.001199 (Figure 4f), and ten yielded 0.00299 (Figure 4g). Sequential measurements crossing the plume border and capturing measurements above and below the detection threshold clearly delineate the shape of the belief, separating regions of high and low probabilities. If the belief was updated with the five measurements at the same time, this would result in an identical belief shape and entropy reduction (Figure 4h), as the scenario where the belief is updated sequentially. Scenario 6, involving two crossflow sections at different distances (Figure 4i), shows patterns similar to Scenario 5, with distinct low-probability regions at measurement locations and increasing probability in an inverse Gaussian shape in the upflow direction. The entropy reduction value of 0.0029 closely matches the single-crossflow scenario with ten measurements from Scenario 5. In Scenario 7, the diagonal crossflow movement (Figure 4d) gains information (0.00107), similar to crossflow sequences. However, varying the movement angle showed decreasing entropy gains with increased angles, except when obtaining very high-concentration measurements, where entropy reduction increases significantly.

### 4.2. Future Entropy Reductions

**Figure 6:**
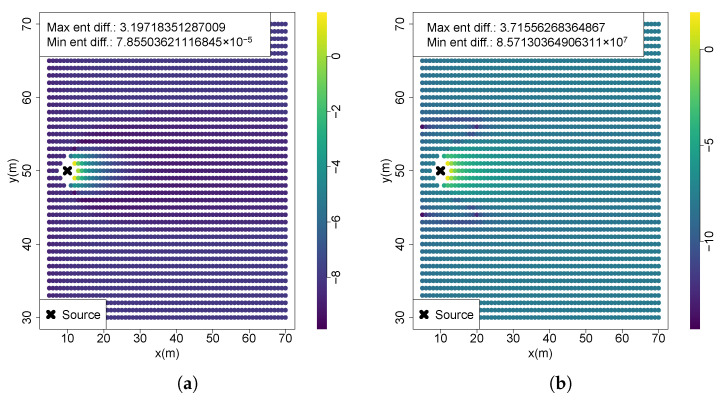
Entropy reduction computations. (**a**) S1; (**b**) S4.

Analyzing entropy reduction across the whole searching space yields approximately the same results at many of the testing scenarios. The reason for this lies in a smaller difference of uncertainty reduction between tested locations, with the positions in the vicinity of source having an extremely higher value than the remaining ones. Hence, the values being presented in logarithmic scale. This can be observed in the computations of the first scenario, shown in Figure 6a with most of the searching area yielding low values, and only a small region near the source with significantly higher uncertainty reductions (the minimum entropy difference is $7.855\times 10^{-5}$ while the maximum is 3.197). The gain in information gradually diminishes in the downflow direction. This situation is also identical to Scenarios 2, 3, 5, 6 and 7, which is related to the fact that all these scenarios have a highly uncertain belief that when used as a prior for future entropy computation, also originates an uncertain future belief and, as a consequence, a lower uncertainty reduction. Locations near the source yield exponentially higher chemical concentrations, substantially reducing future belief uncertainty and resulting in significantly greater entropy reductions. Scenario 4, however, utilized a belief map updated with a high-concentration measurement closer to the source, thus starting from a less uncertain state. As depicted in Figure 6b, regions immediately downstream of the source maintain the highest entropy reductions. Nevertheless, within the active plume region, numerous locations exhibit relatively high entropy reductions, whereas crossflow sections outside the plume region show lower information gains. Consequently, in this scenario, the regions of highest informativeness align closely with the plume’s spatial distribution.

Studying the gains in information on all of the searching space does not provide meaningful conclusions because the gains in information near the source dominate the remaining regions. However, a searching agent only evaluates a small number of locations in its vicinity and is guided by the differences between the gains of information (even small ones, as the highest difference determines the next movement location). So, shifting the study towards individual and local cross-flow sections of the plume provides more insightful conclusions where patterns of information gain start to emerge.

**Figure 7:**
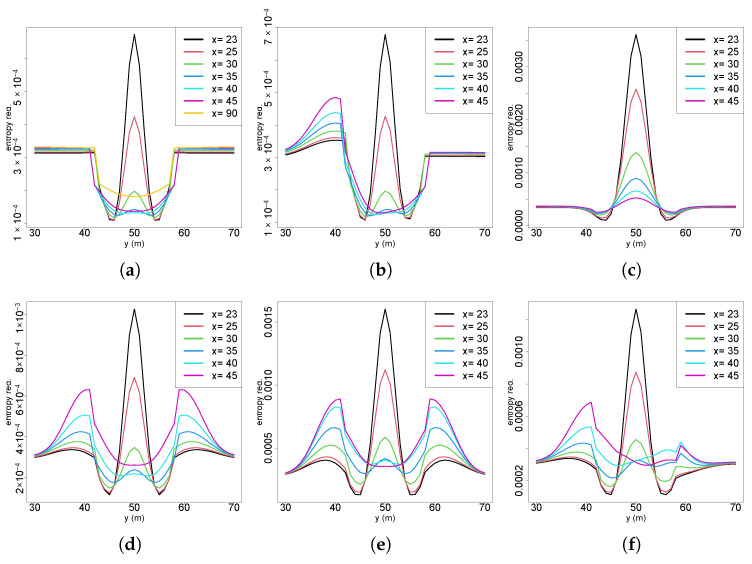
Two-dimensional perspectives of future entropy reductions: (**a**) S1; (**b**) S2; (**c**) S4; (**d**) S5.2; (**e**) S6; (**f**) S7.

Figure 7a shows the 2D perspective of future entropy reductions across multiple crossflow sections of the plume in Scenario 1, where the x-axis represents crossflow (y) coordinates, and the y-axis indicates entropy reduction. Outside the active plume region, entropy reductions are consistently similar and slightly higher at farther sections (e.g., x = 45). Upon entering the active region, entropy reductions initially decrease. However, closer to the source (crossings at x = 25 and x = 23), central plume locations exhibit higher information gains compared to outside regions. This general trend appears consistently across all scenarios, though specific variations emerge depending on the initial belief conditions. In Scenario 2 (Figure 7b), a clear distortion occurs on the side of the plume near the previous measurement location, coinciding with a region of lower probability caused by a measurement below the detection threshold. This distortion diminishes in the upflow directions, whereas higher information gains persist near the plume centerline due to higher chemical concentrations and thus greater surprise and information gain. Scenario 4 (Figure 7c) reveals inverse patterns with lower information gains outside the active plume region, with progressively increasing entropy reductions within the plume towards the source. This scenario, which is characterized by a lower initial uncertainty originating from an intense chemical detection near the source, confirms that closer crossflow sections consistently yield higher gains in information. In Scenario 5.2 (Figure 7d), bilateral distortions appear prominently due to multiple measurements crossing the entire active region. A notable result is the farthest crossflow section displaying higher gains near the centerline than some upflow sections. This phenomenon arises from interactions between the prior probability distributions and future posterior belief updated from the expected measurements, highlighting the influence of Bayesian inference dynamics in future entropy reduction calculations. Similar findings occur in Scenario 6 (Figure 7e), where farther crossflow sections downflow of the measurement positions that updated the belief have notably lower entropy reductions compared to nearer ones. Scenario 7 (Figure 7f) exhibits an asymmetric distortion despite multiple diagonal plume crossings used to update the initial belief. This asymmetry stems from a higher frequency of chemical observations with no odor detection at the initial locations of the diagonal crossing path compared to later positions.

### 4.3. Plume Border Analysis

**Figure 8:**
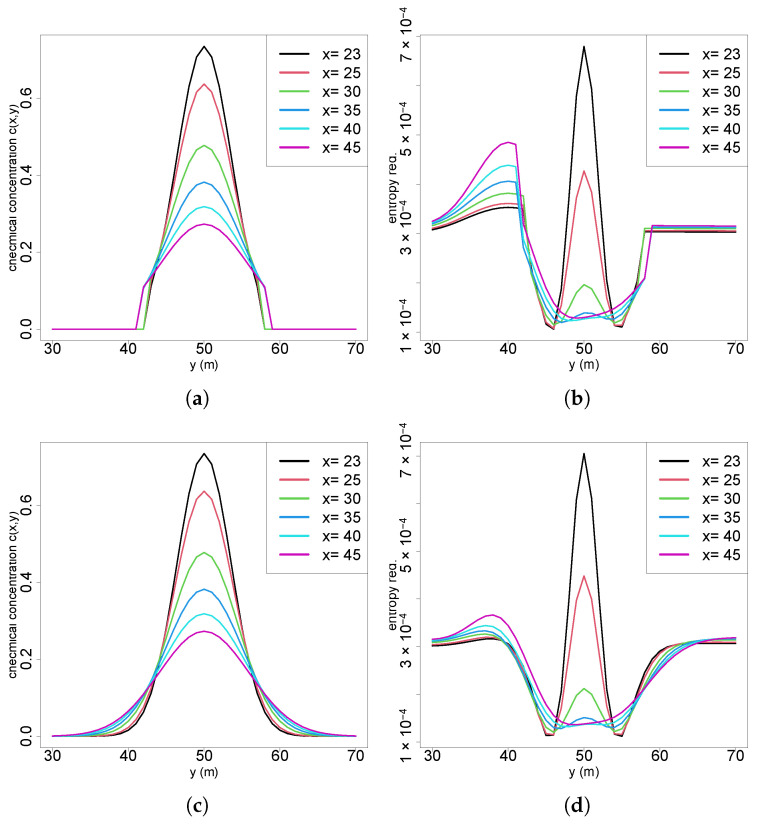
(**a**) Chemical concentration at crossflow sections; (**b**) entropy reduction at crossflow positions of Scenario 2; (**c**) chemical concentration at crossflow sections without detection threshold; (**d**) entropy reduction at crossflow positions of Scenario 2 without detection threshold.

In several of the earlier scenarios, regions near the plume boundaries exhibited distinctive patterns characterized by abrupt shifts in entropy reduction, warranting closer examination. Analysis of the concentration profiles in the crossflow sections (Figure 8a) reveals a steep gradient in chemical concentration between y=40 and y=43 and symmetrically on the opposite side between y=57 and y=60. In these intervals, the concentration sharply increases from 0.0 (no odor detected) to 0.1, corresponding to the detection threshold used in the calculations. Any measurement below this threshold is treated as zero, which shows the influence on the entropy computations. When comparing these concentration gradients to the entropy difference curves (Figure 8b), it shows that the fluctuations in entropy reduction align closely with these threshold-crossing regions. This correlation suggests that the sharp transitions in chemical concentration near the plume boundaries contribute directly to localized spikes in entropy reduction.

When the odor detection threshold is removed, as illustrated in Figure 8c, the abrupt increase in chemical concentration at the plume borders is no longer present. Instead, the concentration profile follows a smooth Gaussian distribution, consistent with the Gaussian plume model. By similarly eliminating the threshold in the likelihood calculations for future entropy reduction, it can be observed in Figure 8d (Scenario 2) that the entropy reduction evolves more smoothly and closely aligns with the plume shape. Notably, the distortion on the left side of the plume observed in the original scenario is significantly attenuated. While the odor threshold introduces distortions in information gain, it is a necessary mechanism in real-world applications to differentiate genuine chemical signals from environmental noise. Therefore, it was retained in all analyses presented.

**Figure 9:**
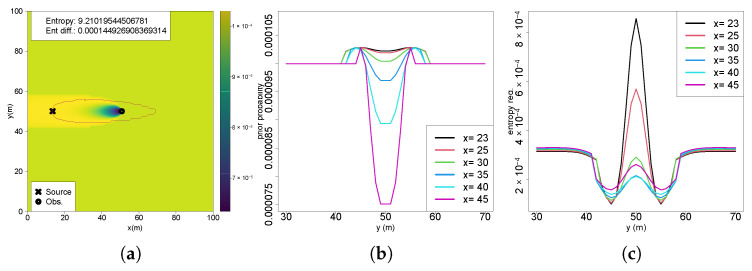
(**a**) Prior belief map of the third scenario; (**b**) probability values of the belief map from the third scenario at each cross-flow position; (**c**) entropy reduction in Scenario 3.

Although the impact of the odor threshold on information gain is evident, it does not fully explain the complex spatial patterns observed. One of the patterns consists of entropy reduction fluctuation near the plume boundaries under highly uncertain priors, while another consists of the monotonically increase of entropy reductions towards the plume centerline under more certain priors. The explanation lies in the Bayesian inference framework. The posterior probability distribution is determined by the product of the prior and the likelihood of the observed data. Entropy, as a measure of uncertainty, is computed from this posterior. In Scenario 2, which begins with a highly uncertain prior (Figure 4a), regions near the measurement location, where the posterior probability of source presence is low, correspond to high information gain (Figure 8b). As it moves upflow and the prior probability increases, the gain in information decreases, revealing an inverse relationship. A contrasting case is shown in Scenario 3 (Figure 9a), where the measurement is obtained inside the plume but farther from the source. Here, the probability distribution is inverted relative to the plume, with lower probabilities near the measurement location and higher probabilities upflow. Cross-sectional analysis (Figure 9b,c) reveals a clear anti-correlation: probability distributions mirror the plume shape, while entropy reduction exhibits an inverse trend. This supports the idea that high likelihood values at future positions increase the magnitude of entropy reduction, as predicted by Bayesian inference. Importantly, information gain is not determined solely by the prior probability at a specific position. It results from the change between the entire prior and posterior probability maps.

Another explanation is related to the concept of surprise from spatial information theory. Observations that are rare (i.e., have low prior probability) produce higher surprise and, consequently, greater information gain. For instance, in Scenario 2, an observation outside the plume results in a low-probability region in the posterior, leading to high information gain. In contrast, within the plume, especially with low concentration readings, similar values can occur at many positions, increasing posterior uncertainty. On the other hand, high concentration readings near the source are rarer and produce more surprise, resulting in higher information gains. Thus, the spatial patterns of entropy reduction are jointly influenced by the chemical plume distribution and the shape of the prior probability map. These patterns reflect the non-linear interaction between prior belief uncertainty, likelihood of observations, and posterior update in Bayesian inference.

**Figure 10:**
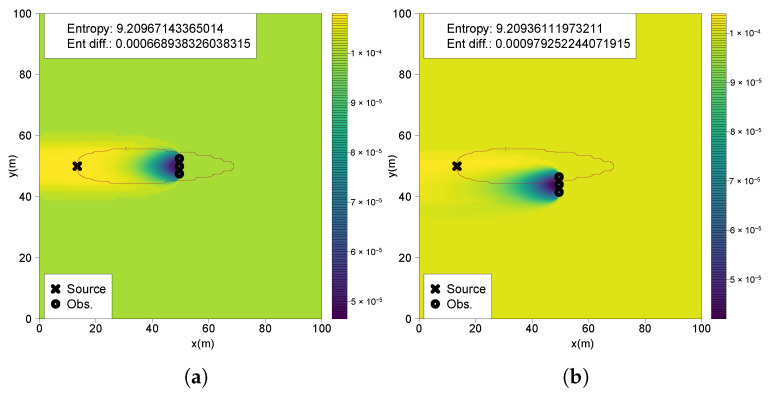
(**a**) Resulting belief from measurements inside the plume; (**b**) resulting belief from measurements crossing a plume border.

To complete the previous analysis, another experiment is performed with two crossings: (1) inside the active plume region with three positive odor detections without trespassing a plume border (0.2, 0.35, 0.2), (2) on a single plume border with one measurement without a positive odor encounter and two measurements above the detection threshold (0.0, 0.15, 0.25). Notably, the measurements collected along the plume border (Figure 10b) result in a higher total information gain (0.000979) than those obtained entirely within the active plume (Figure 10a) (0.00066). This observation implies that, under conditions of high prior uncertainty and considerable distance from the source, a sequence of measurements crossing a plume boundary can be more informative than a sequence taken solely within the active region. This highlights the strategic value of boundary-crossing observations in the early stages of a source localization mission.

### 4.4. Spatial Entropy Analysis

**Figure 11:**
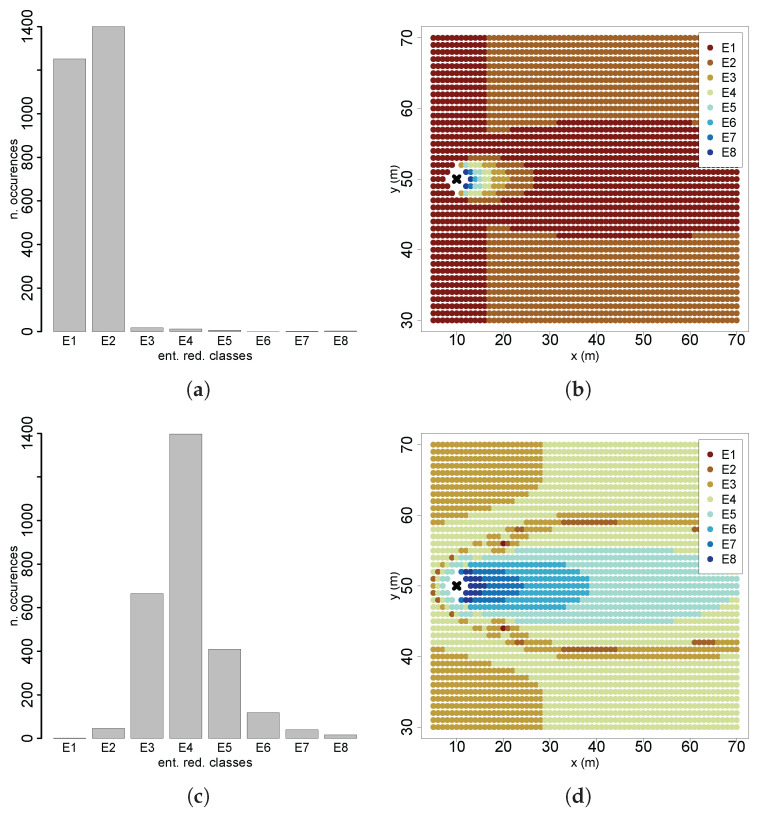
Distribution of entropy reduction classes: (**a**) S1; (**c**) S4. Spatial distribution of entropy reduction classes: (**b**) S1; (**d**) S4.

The results from the spatial entropy analysis provided further conclusions on the patterns of information gains by quantifying the influence of spatial distribution on total entropy values. In Scenario 1, entropy reduction classes predominantly consisted of low information gains, with high-gain classes significantly less represented (Figure 11a). Spatial mapping of these classes (Figure 11b) revealed that higher information gains are concentrated near the source, decreasing gradually downstream. The lowest information gain class formed a spatial pattern closely resembling the plume shape, whereas regions outside the active plume area, especially farther from the source, exhibited relatively higher information gains. This spatial arrangement provides insight into crossflow movements, which are typical during the initial stages of cognitive searches. Scenario 4 presented the opposite situation, with higher-information-gain classes more prominently represented (Figure 11c). These higher gains were spatially aligned with the active plume region, closely following its shape (Figure 11d). These findings highlight that when initial beliefs are highly uncertain and the agent is far from the source, higher future information gains are primarily located either outside the plume (near the plume borders) or close to the source. Conversely, when initial beliefs are more certain, higher future information gains align closely with the active plume region. Thus, both plume geometry and chemical concentration significantly influence information gains, justifying exploratory behavior early in the search through crossflow trajectories and increasingly directed movements as uncertainty decreases.

**Figure 12:**
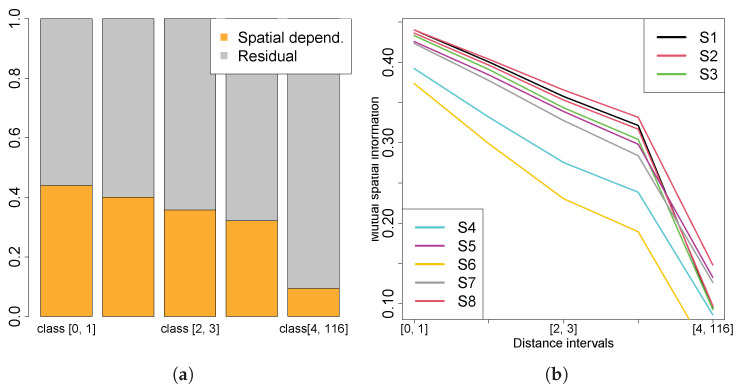
(**a**) Spatial entropy from Scenario 1; (**b**) spatial mutual information from all scenarios.

Analysing the spatial entropy information in Scenario 1 (Figure 12a) indicates that, for lower distance classes, between 40% and 45% of the entropy is explained by spatial dependencies, with the remaining 58% originating from other sources of information. This indicates that entropy reductions are spatially clustered at small scales, with nearby cells yielding similar reductions in uncertainty, which corroborates with previous conclusions. It also represents that the information gain in a cognitive OSL strategy is highly local, with sensor observations impacting the belief update mostly in its immediate vicinity. As the distance intervals increase, spatial mutual information decreases, which indicates less dependence on spatial configurations. In the larger interval, the influence from spatial configurations on entropy reductions is less significant, which indicates a limited interaction between the gains in information over large areas. This means that information gained in a certain region does not significantly influence information gains in distant areas. These results are particularly relevant, as the agent bases its decisions primarily on local information gains, and therefore, higher spatial dependence at shorter intervals implies that agent decisions are strongly influenced by spatial configurations. Since the prior belief is uniform, this also suggests the existence of a localized exploration process with the agent more informative in its local vicinity.

Figure 12b resumes spatial mutual information (proportion of spatial dependency relative to total entropy) for all scenarios across different distance intervals. The x-axis values range from one (smallest distance interval) to five (the largest). These results indicate that the observed trends are consistent across all test scenarios, underscoring the presence of spatial dependence in entropy reduction. Scenarios initialized with higher certainty (i.e., lower entropy) tend to exhibit lower spatial mutual information, reflecting weaker spatial dependencies. In contrast, scenarios beginning with higher uncertainty show greater spatial dependence, which is expected given that high information gain typically occurs near the source. This suggests that variations in entropy reduction are strongly influenced by spatial phenomena. In a cognitive OSL scenario, the spatial distribution is governed by the chemical plume, since it provides the observations to update the belief of the source parameters, and the plume model, which provides the means to compute the likelihood of the observations by relating the chemical concentration with the source parameters.

## 5. Conclusions

This work studied the spatial distribution of entropy reductions along odor searching processes. The aim was to identify informative patterns that could be leveraged to design effective OSL strategies. The study assumed a Gaussian plume model for odor dispersion. The results highlighted the critical roles of the plume model and the agent’s belief state in shaping information gain. Regions near the source consistently yielded the highest entropy reductions, often orders of magnitude greater than the remaining regions.

In conditions of high belief uncertainty and far from the source, the difference between entropy reductions in nearby regions is minimal, which suggests similar gains in information regardless of the decision and justifies why cognitive agents often engage in extensive exploration to significantly reduce the uncertainty of the belief. Nevertheless, entropy differences still exist, with the most informative regions identified across the plume borders and centerline, indicating that crossflow movements perpendicular to the plume axis yield higher information gains during these early search stages. As uncertainty decreased, highly informative regions became concentrated within the plume itself, taking a shape similar to the active plume area. These findings show that entropy reduction is influenced both by the prior belief and the predicted posterior from future observations. The conclusions from these patterns suggest cross-movements during the first stages of the search when the agent is far from the source and the uncertainty of the belief is higher. As the knowledge of the belief increases, the motion shifts towards more directed, or exploitative decisions, resulting from the proximity of the most informative positions being near the source. These tendencies resemble to bio-inspired behaviors such as the ones produced by the silkworm moth, performing crossflow zigzag patterns when searching for chemicals or when loosing contact with the plume, which occurs more often when it is far from the source, and surge movements in the upflow direction when detecting the odorant, more frequent when near the source.

The previous results were validated by a spatial entropy analysis which confirmed that cognitive decisions, which are local, depend strongly on spatial characteristics of the environment, particularly the chemical observations and plume dispersion model. Importantly, this analysis revealed that plume boundaries serve as valuable constraints for bounding search movements, as agents can navigate within the active plume region without necessarily detecting the chemical signal due to intermittency and turbulent fluctuations. This insight suggests that maintaining search patterns within these high-information boundaries, rather than relying solely on instantaneous chemical detections or moving between the limits of the search area, can significantly enhance search efficiency by preventing unnecessary exploration of low-information regions while accounting for the stochastic nature of odor encounters. These insights provide a foundation for synthesizing hybrid search strategies that retain the effectiveness of cognitive decisions while significantly reducing the computational cost following the strengths of bio-inspired behaviors.

## Data Availability

The original contributions presented in this study are included in the article. Further inquiries can be directed to the corresponding author.

## Abbreviations

The following abbreviations are used in this manuscript:

OSL	Odor Source Localization
PDF	Probability Density Function
